# *Malus sieversii*: a historical, genetic, and conservational perspective of the primary progenitor species of domesticated apples

**DOI:** 10.1093/hr/uhae244

**Published:** 2024-08-30

**Authors:** Richard Tegtmeier, Anže Švara, Dilyara Gritsenko, Awais Khan

**Affiliations:** Plant Pathology and Plant-Microbe Biology Section, School of Integrative Plant Science, Cornell University, Geneva, NY 14456, USA; Plant Pathology and Plant-Microbe Biology Section, School of Integrative Plant Science, Cornell University, Geneva, NY 14456, USA; Laboratory of Molecular Biology, Institute of Plant Biology and Biotechnology, Al-Farabi Kazakh National University, Almaty 050040, Kazakhstan; Plant Pathology and Plant-Microbe Biology Section, School of Integrative Plant Science, Cornell University, Geneva, NY 14456, USA

## Abstract

Apples are one of the most valued tree fruit crops around the world. Currently, a few highly popular and economically successful apple cultivars dominate the commercial production and serve as main genetic contributors to the development of new apple cultivars. This limited level of genetic diversity grown as a clonally propagated monoculture renders the apple industry vulnerable to the wide range of weather events, pests, and pathogens. Wild apple species are an excellent source of beneficial alleles for the wide range of biotic and abiotic stressors challenging apple production. However, the biological barriers of breeding with small-fruited wild apples greatly limit their use. Using a closely related wild species of apple such as *Malus sieversii* can improve the efficiency of breeding efforts and broaden the base of available genetics. *M. sieversii* is the main progenitor of the domesticated apple, native to Central Asia. The similarity of fruit morphology to domesticated apples and resistances to abiotic and biotic stresses makes it appealing for apple breeding programs. However, this important species is under threat of extinction in its native range. Preserving the wild apple forests in Central Asia is vital for ensuring the sustainable protection of this important genetic resource. The insufficient awareness about the complete range of challenges and opportunities associated with *M. sieversii* hinders the maximization of its potential benefits. This review aims to provide comprehensive information on the cultural and historical context of *M. sieversii*, current genetic knowledge for breeding, and the conservation challenges of wild apple forests*.*

## Introduction

Apples (*Malus domestica* Borkh.) are a highly valued tree fruit crop globally contributing an estimated $73 billion to the global economy [1]. The culinary appeal, deep cultural connection, and human health benefits drive the popularity of apples and the desire to preserve the crop. Thousands of apple varieties were grown worldwide to produce high-quality fruit for the fresh market, a range of beverages, and processed food products [2]. However, within the last century, there has been a steady decline in the diversity of apples used for breeding and commercial production [3]. Despite the robust genetic resources available, presently, a handful of economically successful apple cultivars make up most apples produced and background genetics of new apple cultivars [3, 4]. In modern apple production, more than half of all commercially US produced apples consists of only four apple cultivars, namely “Red Delicious”, “Gala”, “Fuji”, and “Honeycrisp” [5, 6]. The background genetics of many of these economically successful cultivars stem from a narrow base of ancestral cultivars such as “Golden Delicious”, “Cox’s Orange Pippin”, “Jonathan”, “McIntosh”, and “Red Delicious” [3]. The limited number of cultivars and genetic diversity employed in large monocultured systems renders the apple industry vulnerable to disruption from a wide range of weather events, pests, and pathogens [4, 7]. To alleviate this risk of disruption, harnessing of closely related crop wild relatives is an effective method for enhancing genetic resistance to biotic or abiotic stresses [2].

*Malus sieversii* (Ledeb.) M.Roem, the primary progenitor of domesticated apples, represents a key focal point for broadening genetic diversity and introducing beneficial loci into apple breeding programs [8]. *M. sieversii* is a well-studied wild apple, native to Central Asia with the most similar fruit morphology to domesticated apples [9]. *M. sieversii* has a plethora of beneficial traits associated with resistance to abiotic and biotic stressors that have been characterized [10]. Nevertheless, the conservation status of this invaluable genetic resource in Central Asia is categorized as vulnerable [11]. Threats of climate change, genetic erosion, and habitat degradation will require international level action to help preserve the wild apple forests in this region [12]. A better understanding of the complete range of challenges and opportunities associated with *M. sieversii* can help maximize its potential benefits. This review aims to provide comprehensive information to the cultural and historical context of *M. sieversii*, current genetic knowledge relevant to breeding, and the challenges of wild apple forest conservation*.*

## Domestication from *M. sieversii* to *M. domestica*: the historical and cultural context

### Origins and early documentation of *M. sieversii*

The journey from wild apples to their domesticated counterpart begins in the geographically diverse region of Central Asia [2, 9]. More specifically, the center of origin of the domesticated apple is in the Tian Shan mountains of modern-day Kazakhstan [2, 9, 13]. The establishment of this region as the origin started with the first formal documentation of the wild apple forests in this region by the German botanist, Johann August Carl Sievers (1762–1795) [14]. As a prominent member of the Russian Imperial Academy of Sciences, Sievers embarked on an expedition to Siberia and Central Asia in 1793 [15]. Sievers recorded his observations of the fruit from the wild apple forests in the infamous “eleventh letter” as apples the size of chicken eggs with red and yellow cheeks [14]. To honor Sievers’ contributions to this field, the German botanist Carl Ledebour coined the species name *sieversii* in his most prominent publication, *Flora Altaica* [16]. By the early 20th century, the Russian botanist Nikolai Ivanovich Vavilov was documenting the first evidence of the ancestral connection of *M. sieversii* to the domesticated apple [13]. Vavilov traveled through Central Asia, reporting the highest levels of apple diversity to be in southeast Kazakstan near a city called Almaty (previously Alma-ata; “Father of apples”) [13]. The fruit in these forests also bared the greatest similarities with domesticated apples above all other wild crabapples he observed [13, 17, 18]. Vavilov concluded this was the center of origin of the domesticated apple, which was later strengthened with evidence from genetic markers and sequencing data [19–21]. It is now well established that *M. sieversii* is the main progenitor species of modern apples [9, 22, 23].

### Evolution and domestication of *M. sieversii*

*M. sieversii* belongs to the family *Rosaceae* that encompasses many popular edible and ornamental crop plants, including rose, pears, cherries, peaches, strawberries, and almonds [24]. In addition to these crops, *Rosaceae* includes 100 genera and nearly 3000 species [25, 26]. This family was originally divided into four sub-families: *Amygdaloideae*, *Maloideae*, *Rosoideae*, and *Spiraeoideae* [25–27]. *Rosaceae* has since been consolidated into a three sub-family division of *Amygdaloideae, Rosoideae*, and *Dryadoideae* [25–27]. These initial classifications were driven by distinctions such as chromosome numbers and ovary position of *Maloideae* (2*n* = 34; inferior ovaries) compared to *Amygdaloideae* (2*n* = 16, 18; superior ovaries) [27]. With an increase in available genomic data, the species forming *Maloideae* were grouped into *Amygdaloideae* and renamed *Maleae*, the apple tribe [27]. *Maleae* includes many other slow-growing woody perennial plants adapted to temperate climates [28, 29]. The evolution of *Maleae* was driven by a series of polyploidy events, point mutations, whole genome duplications (WGDs), and interspecific hybridizations that generated a great level of diversity amongst large pomes in the *Malus* clade [19, 21, 28]. Previous genome analyses revealed a WGD event shared by apple, pear, *Sorbus*, loquat, and hawthorn, members of the *Maleae* tribe [21, 29, 30]. This contributed to the divergence of *Malus* from its close relative, the pear in the *Pyrus* genus ~8–16 million years ago [28]. These WGD events gave the members of *Maleae* the characteristic haploid set of 17 chromosomes from the ancestral set of 9 haploid chromosomes [21, 28]. The generation of new gene copies from these duplication events likely favored the diversification and adaptation of *Maleae* when facing dramatic shifts in climate [28].

**Figure 1:**
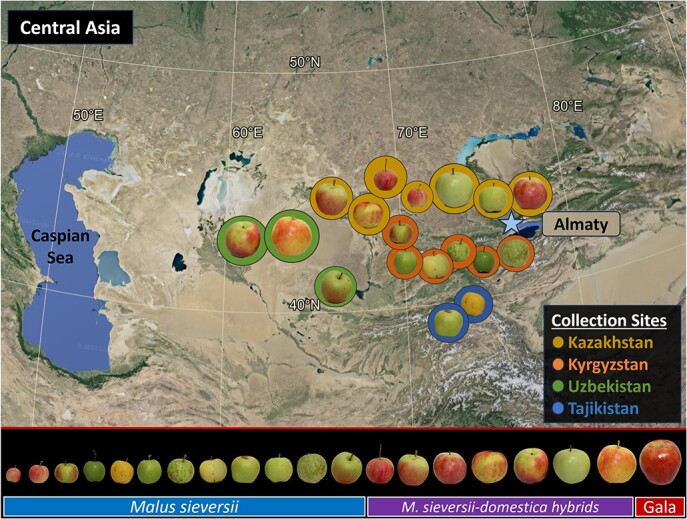
A geographical map of Central Asia showing in color the regions where *Malus sieversii* (Ledeb.) M.Roem is native, not including Afghanistan. A small subset of *M. sieversii* and *M. sieversii–M. domestica* hybrid apples are placed in the approximate regions where the United States Department of Agriculture—Plant Genetics Resource Unit first collected samples. The apples on the map are scaled relative to each other to compare fruit sizes. The scale on the bottom row gives a reference for each fruit size relative to *M. domestica* cv. “Gala” on the far right of the scale. The species classifications are assigned according to Volk et al. [35].

*Malus* encompasses over 30 species of wild, landrace, and fully domesticated apples, growing across both the Northern and Southern temperate zones [9]. The distribution of these *Malus* species is spread across every continent except Antarctica primarily in Northern Asia, Europe, and North America [1, 2]. The evolution of the ancient *M. sieversii* populations was mainly driven by different modes of seed dispersion, geography, and self-incompatibility of apple [31, 32]. Selection and dispersion by early humans, birds and other mammals for fruit quality and size, primarily caused the diversification of fruit morphology [32, 33]. *M. sieversii* is known for fruit with a substantially larger average diameter comparable to modern cultivars (Fig. 1). The fruit diameter can range from approximately 4 cm to 6 cm [18], up to 7 cm [34] compared to other known wild crabapples. Though some apples from these studies could be a result of hybridization with *M. domestica*, there are pure *M. sieversii* accessions such as “Ketmen Dessert” with an average 6.3 cm fruit diameter [35, 36]. This suggests that megafaunal dispersal of *M. sieversii* seeds was more likely than avian dispersal [32, 37]. Megafaunal dispersion is far more limited in range than avian dispersion, suggesting human intervention was a key factor in the eventual spread of *M. sieversii* out of Central Asia [32, 37]. Grafting techniques developed in the Neolithic era were also essential towards enabling wider distribution through the cloning and exchange of superior apples [22, 31]. Despite increased selection intensities of domestication and cloning of favorable genotypes, apples maintained a moderate level of genetic diversity and have avoided major bottlenecks [22, 38]. Ultimately, human intervention played a pivotal role in establishing wild populations of large-fruited *M. sieversii* apples across Central Asia [32].

Wild standings of *M. sieversii* that can be found across a wide area in Central Asia including Kazakhstan, Kyrgyzstan, Tajikistan, Uzbekistan, Turkmenistan, and the western part of China [9, 21, 39]. Notably, along the Trans-Ili Alatau section of the Tian Shan mountains stretching from southeastern Kazakhstan to Kyrgyzstan [40]. The Tian Shan mountains acted as a unique natural protection for *M. sieversii* whereas most other wild apple populations were found in glacial refugia zones [32]. Tectonic plate shifting and glacial deposits across the mountainous areas have generated a fertile and well-suited soil for the growth of these wild apples [41, 42]. Beyond these mountains, *M. sieversii* grows in an extremely diverse set of geography including environments that are cold and mountainous, humid-temperate mixed forests, and xeric, mixed scrub forest [4]. Adaptation to these range of environments resulted in the variation in growth habits, fruit quality, and environmental resilience that makes *M. sieversii* so valuable [4, 34].

Several important subgroups of *M. sieversii* comprise the wild populations found across geographically diverse regions of Central Asia and Northern-Western China. *M. sieversii* from far western Xinjiang, China with characteristics similar to domesticated apple were found to be an ancient, isolated ecotype not directly contributing to apple domestication [19, 43]. *M. sieversii* in Xinjiang has retained high intraspecific homology with the lowest levels of heterozygosity compared with *M. sieversii* in Kazakhstan with relatively high heterozygosity [19]. Additional *M. sieversii* subgroups native to Central Asia include *M. sieversii var. kirghisorum, M. sieversii var. turkmenorum*, and *M. sieversii f. niedzwetzkyana* [41, 44]*.* Over time, these subgroups have been placed in a wide range of botanical classes such as independent species, subspecies, varietals, and forms likely due to the distinct morphological differences from *M. sieversii*. For example, Dzhangaliev et al. [41] classified the red fleshed *M. sieversii* apples as *M. niedzwetzkyana* based on morphological observations. Volk *et al*. [44] with limited SSR marker data found evidence of highly overlapping gene pools between these red fleshed variants previously characterized as *Malus pumila var. niedzwetzkyana* and accessions labeled *M. sieversii*, *M. sieversii var. turkmenorum, M. sieversii var. kirghisorum,* and *M. pumila*. More in-depth genetic analyses support that these groups are not discernable of outside of a common taxon of *M. sieversii* [35, 44–46]. Limited studies suggest that *M. sieversii var. turkmenorum* native to Iran and Turkmenistan could be more related with the Caucus apple *M. orientalis* [47–49]. DNA fingerprinting studies reveal that the gene pools represented by all these subgroups are highly overlapping with *M. sieversii* and do not support classification as a unique and independent species [35, 44–46]. Volk *et al.* [44] showed among these subgroups only 2%–8% of the genetic variation observed explains the differences among accessions. The evidence currently suggests that *M. sieversii* is genetically diverse with a wide range of phenotypically distinct subgroups species [35, 44–46].

When the apple genome was first sequenced [21], new advances were developed in the understanding of the evolution and domestication of *M. sieversii*. Comparative analyses of resequencing data showed *M. sieversii* was less closely related to *M. sylvestris, M. baccata, M. micromalus,* and *M. prunifolia*, whereas *M. orientalis* and *M. asiatica* show genetic similarity to *M. sieversii* [19, 20]. Similar findings of the contributions of *M. sieversii* to domesticated apples have been shown with molecular marker data [35, 50]. However, a greater contribution of *M. sylvestris* has been found from maternal inherited chloroplast genomes [51]. The availability of sequencing data has helped add a more refined insight of the process from wild *M. sieversii* apples into the cultivated apple along the ancient Silk Road [19, 20].

The Silk Road was a 4000-mile network of trade routes that connected China with Central Asia, South Asia, the Middle East, Turkey, and Europe between 130 B.C. and 1453 A.D. [52, 53]. The apples were carried from Central Asia along the Silk Road trading routes through merchant caravans from the beginning of the Neolithic period through the Bronze Age [54]. The apples brought from Central Asia would be eaten and tossed along the path where seeds could germinate, colonize the local area, and hybridize with local wild apple species enabling wider dispersion of their genetics along these roads [20, 54]. Apples have been found to have several important centers of genetic diversity that contributed to the speciation and diversification of the genus. These include Europe, North America, Central Asia, Central China, and the Caucus region [4]. The admixture of *M. sieversii* with several other wild *Malus* species along the Silk Road, including *M. sylvestris* (Europe), *M. orientalis* (Caucus), and *M. baccata* (Siberian) resulted in genetic contributions to the gene pool of modern apples [9, 20]. From the Middle East and contact with the Persians, the Greeks and Romans brought apples into Europe, about 1500 years ago, through trade in the Mediterranean [9]. Among the wild apples of Europe is where *M. sieversii* hybridized with the European crabapple *M. sylvestris*, making important secondary contributions to the gene pool of domesticated apples [9, 22, 23]. The economic value of these large wild apples on the Silk Road was a driver of their distribution westward and hybridization with other *Malus* species [53].

### Cultural and symbolic heritage of apples

Over the course of history, civilizations along the Silk Road and beyond have embraced the apple as a cultural symbol, incorporating it into their traditions and heritage [55]. As early as 200 BCE, Central Asian and Chinese communities were utilizing *M. sieversii* apples not only for food and fermented alcoholic beverages, but as medicinal remedies as well [56]. Dried fruit such as wild apples, bread, and milk have been the staple diet of many central Asian countries and nomadic tribes for centuries [57]. Traditionally, in many central Asian countries such as Tajikistan fruits are a revered gift or prize to be eaten at celebrations and major ceremonies [57]. For example, apples in Tajik rituals are a symbol of birth and new life eaten during the naming of newborn children [57]. This symbolism also extends into Middle Eastern culture as depicted in the famous stories of “The Arabian Nights,” where the magical apple is portrayed to cure any illness and restore vitality [58]. In the Bible, the apple eaten by Eve is a symbol of temptation and desire possibly sparked from the Latin name *Malus* also meaning “evil” [33, 59, 60]. In Norse and Greek mythology, the “golden apple” provided the gods with immortality [60]. Similarly, wild apples are symbol of pride and identity for Kazakhstan [61]. To this day, the Almaty apple festival in Kazakhstan celebrates the city’s rich history and diversity of apples at center of origin for the domesticated apple [62]*.*

In the modern global trading routes, a few *M. domestica* apples now make up the vast majority of fruit produced, traded, and used for breeding [4]. Internationally planted cultivars with a more predominant genetic background of *M. sieversii* are not commonly found. The “Aport” (“Alexander”) apple is a key example of how admixed hybrids of *M. sieversii* and *M. domestica* can be a valuable and accessible source of genetic material from *M. sieversii* [63]. “Aport” is large fruited with good quality but interestingly maintains the best graft compatibility with *M. sieversii* rootstocks (Unpublished data). Introduced to Kazakhstan in the mid-19th century by immigrants from the Voronezh province of Russia, “Aport” is a high-quality apple, regarded as a symbol of the culture in the Alatau Mountains near Almaty [63]. Though there are successful regional apple cultivars, international cultivars continue to dominate the global market share of apples [4].

## Biological challenges and opportunities of breeding apples using *M. sieversii*

Wild *Malus* species are a valuable source of useful genetics (Table 1), and the biological challenges of breeding apples favor the use of *M. sieversii* compared to most other wild *Malus* species. The major hurdles of breeding a long-cycle perennial crop such as apple includes a 4- to 6-year juvenile period, high heterozygosity, and gametophytic self-incompatibility [64, 65]. Using wild apple relatives in conventional crossing scheme would require nearly 25 years to effectively break the linkage drag of unfavorable fruit quality alleles [66, 67]. Additionally, making crosses with distantly related apples can suffer from low fertilization efficiency and post-zygotic barriers, such as endosperm abortion, requiring the use of embryo rescue [68, 69]. *M. sieversii* is among the wild *Malus* species that is highly sexually compatible with *M. domestica* while having the most similarly sized fruits to domesticated apples [9, 70]. This would improve the efficiency of apple breeding and require fewer pseudo-backcross generations to recover superior genotypes. In addition, there are more than 20 known self-incompatibility (S) alleles from *M. domestica* [71] and 14 distinct S-alleles from *M. sieversii* [72]. Utilizing *M. sieversii* accessions to diversify the S-alleles present in current breeding programs will help reduce the challenge of incompatibility when breeding within a narrow genetic pool of elite cultivars [72]. This makes it more streamlined to deploy the plethora of known loci from *M. sieversii*, linked with resistance to abiotic and biotic stressors. All these factors together make *M. sieversii* a valuable target for improved apple breeding.

**Table 1:** The alleles of available quantitative trait loci (QTL) or genes from *Malus sieversii* that are usable for apple breeding.

Trait	Allele	Chr	Region	Accession	Citations
*Apple Scab* *Resistance*	*Rvi8*	2	Distal	W193B	128
	*SNR1*	2	Distal	PI 613988	131
	*SNR2*	2	Distal	PI 613988	131
	*ChlR1*	2	Distal	PI 613988	131
	*ChlR2*	2	Distal	PI 613988	131
*Blue Mold* *Resistance*	*qM-Pe3*	3	Distal	PI 613981	109 138
*Fire Blight* *Resistance*	*Msv_FB7*	7	Distal	PI 613959	118
*Fruit Texture*	*PG1*	10	Proximal	Conserved	20 110
*Fruit Weight*	*fw1*	15	Distal	Conserved	19 114
	*fw2*	8	Distal	Conserved	19 114
*Fruit Acidity*	*Ma*	16	Proximal	PI 613988	95 98
	*Ma3*	8	Proximal	PI 613988	9598
*Red Flesh*	*MdMYB10*	9	Distal	Conserved	8891

For each trait in the left column there is the locus name, the chromosome, the general genomic region of the locus, the donor accession name or plant introduction (PI) number, and relevant citations. Where the accession name says conserved the allele was identified to be highly conserved across several *M. sieversii* accessions and other *Malus* species.

Several large-scale breeding programs have reported using *M. sieversii* and *M. sieversii–M. domestica* hybrid [35] accessions for scion breeding programs for disease resistance [73, 74]. Cultivars that have a notable *M. sieversii* contribution include “Aport” and “Saltanat” [63, 75, 76]. However, to our knowledge, no scion cultivars have yet commercialized on a large-scale from these endeavors. Traits such as disease resistance or abiotic stress tolerance derived from *M. sieversii* are more efficiently being utilized by rootstock breeding programs where fruit quality is not the priority trait [67, 77, 78]. Fazio *et al*. [79] evaluated approximately 500 *M. sieversii* derived seedlings for root traits suitable for rootstock breeding. Although no seedlings were reported to have the dwarfing traits important for modern high-density apple production, many seedlings were observed to have beneficial disease resistance and horticulture traits such as flat branching [79]. Superior genotypes from these evaluations were utilized in the Geneva® rootstock breeding program [79].

## Breeding for fruit quality traits

### Pigmentation of fruit skin and flesh

**Figure 2:**
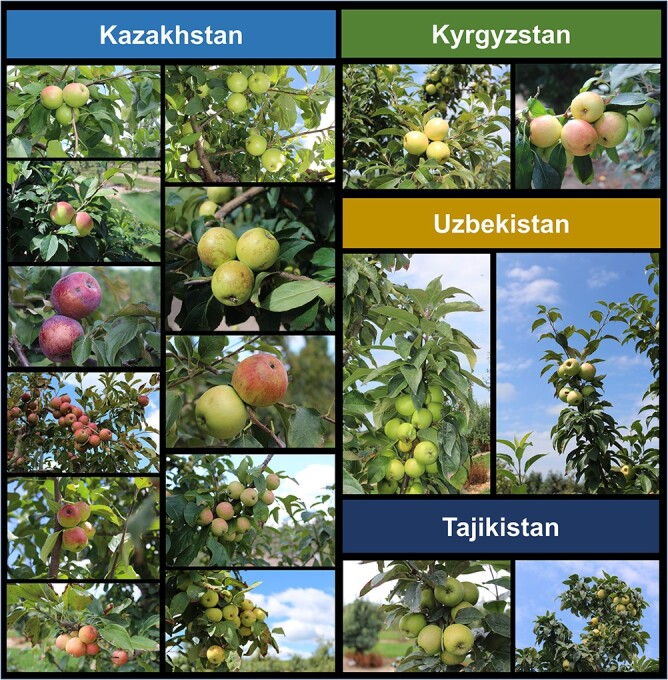
Fruit images of a small set of *Malus sieversii* (Ledeb.) M.Roem and *M. sieversii–M. domestica* hybrid accessions available from the U.S. Department of Agriculture Plant Genetic Resources Unit *Malus* collection in Geneva, NY. The countries in Central Asia from which the accessions were collected are given above the images.

The two primary components of the visual consumer appeal of apple fruit color are the skin and flesh color. A wide range of fruit skin colors can be observed across *M. sieversii* and *M. sieversii–M. domestica* hybrid (Fig. 2)*.* This is controlled by anthocyanin pigment accumulation in the skin and flesh which is affected by genetic and environmental factors [10]. *MdMYB1* and *MdMYBA* are major underlying transcription factors found across *Malus* that control fruit skin color [80, 81]. Molecular markers for selecting this fruit skin color locus are publicly available [81, 82]. Additionally, a retrotransposon (redTE) upstream of *MdMYB1* was observed to upregulate peel anthocyanin content in red skinned apple cultivars [83]. Though the link is not well-characterized, this retrotransposon has been confirmed in resequencing data of red fleshed *M. sieversii* accessions [19, 20, 83]*.* Sun *et al*. [20] showed that this gene was present in the *M. domestica, M. sieversii* genomes and not the *M. sylvestris* genome suggesting *M. sieversii* as the donor. Though the total variation of apple skin color is found in both *M. sieversii* and *M. domestica*, red flesh is a rarer phenotype more prevalent in *M. sieversii* [10].

Red-fleshed apples from *M. sieversii* have been studied for their potential to enhance the health benefits of apples by providing anthocyanins, flavonoids, and other polyphenolic compounds [10]. In contrast to the white-fleshed cultivars from *M. domestica*, red-fleshed apples offer significant phenotypic variation, which can improve both the consumer appeal and nutritional value of newly bred apple cultivars [10]. This work has been particularly focused on the subgroup of red-fleshed genotypes from *M. sieversii* denoted as *M. sieversii f. niedzwetzkyana*, *Malus niedzwetzkyana* Dieck, or Niedzwetzky’s apple [10]. These unique wild apples are native to Central Asia and have a slightly more extensive distribution range stretching south to the Afghanistan border [84]. They were first introduced to the West in 1890 at the Zöschen Arboretum in Germany with seed donated by Vladislav E. Niedzwiecki [76, 85]. These apples were most famously used in the early 20th century by the apple breeder Niels Hansen [76]. Hansen utilized these apples to create new red-fleshed cultivars, the best known of which is “Almata” [76]. Red-fleshed *M. sieversii* accessions and offer great potential to develop apples with good fruit quality and high flavonoid content [74].

The apple flesh color is strongly affected by phenolic compounds, which have been shown to vary mainly in their content across *M. sieversii* [86, 87]*.* Volz and McGhie [86] measured fruit peel and cortical flesh samples from *M. domestica* and *M. sieversii* accessions for flavanol, oligomeric procyanidin, chlorogenic acid, dihydrochalcone, anthocyanin polyphenols, and total polyphenols. The variation of total polyphenol concentration across domesticated and wild genotypes ranged from 7 to 9 fold in the cortical flesh and 4 to 3 fold in the fruit peel [86]. The within-species variation ranged from 2 to 500-fold changes in polyphenols across the different tissue types [86]. *M. sieversii* and *M. sieversii–M. domestica* hybrid [35] accessions have consistently been observed to have a higher total phenolic content than *M. domestica* [70, 86]*.*

Several phenylpropanoid biosynthesis genes control this variation in red flesh. *MYB10* was found to control the anthocyanin pigment accumulation in the flesh [88]. Differential expression of the flavonoid-related genes, *MdbHLH3*, *MdMYB12*, *MdMYB10*, *MdMYB16*, and *MdMYB111* was observed from *M. sieversii f. niedzwetzkyana* red-fleshed fruit at varying stages of development [89]. Wang *et al.* [90] found 22 upregulated flavonoid biosynthesis genes using comparative transcriptomics with an F1 family segregating for the red-flesh phenotype. The *MYB12* transcription factor was observed to interact with the genes *bHLH3* and *bHLH33,* playing a role in proanthocyanin synthesis [91]. *MYB22* was also found to activate flavanols pathways by combining directly with the flavanol synthase promoter [91]. Additionally, Wang *et al*. [92] found *MdBZR1* is an essential component in the accumulation of anthocyanins in the flesh of *M. sieversii f. niedzwetzkyana* apples. These well-characterized genes can be used to select favorable *M. sieversii* accessions [10]. Red-fleshed and red-skinned *M. sieversii* have significant potential as breeding parents to develop apples with higher flavonoid contents, which will benefit consumer health [10, 48].

### Fruit flavor

The major components of apple flavor quality in consumer preference are the balance of acidity and sugar content [65]. Fruit flavor is a highly complex quantitative trait, but the compounds that drive this difference in flavor are mainly malic acid, sucrose, fructose and glucose [65]. Acidity is highly variable among *M. sieversii* and *M. sieversii–M. domestica* hybrid [35] accessions and well-understood on the genetic level. Among the 78 *M. sieversii* and *M. sieversii–M. domestica* hybrid [35] accessions evaluated from Canada’s Apple Biodiversity Collection, *M. sieversii* showed a high level of acidity, but a greater level of variation whereas *M. domestica* was predominantly in the commercially acceptable range [70]. This little variation is likely due to many years of selection on a narrowing genetic pool for apples with malic acid between 3.0 to 10.0 mg/ml^−1^ [70]. Khan *et al*. [93] suggested that when larger fruit size was favored during domestication, selection for low acidity and high sugar content was observed. *Ma* (malic acid) is a major gene encoding a malate transporter discovered in the 1950s [94] and has been shown to be the largest and most conserved contributor to apple acidity [20]. Xu *et al*. [95] found the low-acid trait was primarily controlled by *Ma* on linkage group 16 and segregated recessively in a biparental population of *M. domestica* cv. Royal Gala × PI 613988 (*M. sieversii–M. domestica* hybrid accession [96]). Another major effect locus was mapped to linkage group 8 from a “Prima” × “Fiesta” F1 population [97]. Consistent with these results, Verma *et al*. [98] confirmed the same two major effect quantitative trait loci (QTL), *Ma* (LG16) and *Ma3* (LG8), from a pedigree-connected germplasm consisting of 16 F1 full-sib *M. domestica* families. Sun *et al*. [20] later confirmed with re-sequencing data that the SNP variants in the gene underlying *Ma (MdALMT9* [99]) associated with differences in fruit acidity are highly conserved across the domestication of apples to *M. domestica,* from *M. sieversii,* and *M. sylvestris.* Sun *et al*. [20] found the G to A mutation in the *Ma* gene sequences that results in low acidity [100] at a far higher frequency in *M. sieversii* and *M. domestica* compared to *M. sylvestris*. In concurrence with Davies *et al*. [70] and Watts *et al*. [101] a higher percentage of the *M. domestica* accessions were heterozygous for the *Ma* locus resulting in more optimal pH ranges compared to *M. sieversii* and *M. sieversii–M. domestica* hybrids [35] which had more high acid genotypes, likely due to selection [20]. In addition to the *Ma* genes, Liao *et al.* [102] reinforced the importance of alternate acidity genes, *MdPP2CH* (malate) and *MdTDT* (citrate) conserved between *M. domestica* and *M. sieversii*. Selection of these major effect genes can help efficiently breed more *M. sieversii* derived cultivars with commercially acceptable acidity concentrations [103].

Fruit sweetness plays an important role in the balance of flavor driving much of consumer appeal [104]. Most often fruit sweetness is measured as soluble solid content (SSC) expressed as Brix [70]. *M. sieversii* and *M. sieversii–M. domestica* hybrids [35] accessions across several studies have been found to have no statistical differences in SSC to *M. domestica* cultivars [70, 105]. Li *et al*. [105] showed that phenotyping for specific sugars, namely fructose, glucose, sucrose, and sorbitol, significant differences are observed. Notable genes that have been confirmed in *M. domestica* and *M. sieversii* controlling sugar accumulation in the fruit are *MdWD40* (glucose), *MdSOT2* (sorbitol), *miR172g*, *MdSWEET9b* and *MdSWEET15a* [19, 105–107]. These genes are prime targets for marker assisted selection of *M. sieversii* breeding parents with commercially acceptable fruit sugar content [107].

### Fruit texture, size, and storability

The texture and shelf life of apples are key components to consumer adoption and commercial appeal [65]. *M. sieversii* apples have been reported with a softer mealier texture and short senescence cycles [20, 41]. This can be unappealing to the average consumer with a taste for crisper and firmer apples available year-round [104]. Despite these initial observations, Davies *et al*. [70] found compared to *M. domestica*, *M. sieversii*, and *M. sieversii–M. domestica* hybrids [35] had no statistical differences in percent change of firmness and acidity during storage. Cuticle wax accumulation controlled by *WR1I* contributes to shelf life with differential expression of this gene found in *M. sieversii* and other wild *Malus* species [108]. *M. sieversii* and *M. sieversii–M. domestica* hybrid [35] germplasm has been identified that meets commercial storage requirements and resists storage related diseases such as blue mold [70, 109].

Fruit texture and size are two components of fruit quality focused on breeding programs important to consumer appeal. *Md-ACS1, Md-ACO1, ERF4*, and *Md-PG1* are among several key genes controlling fruit texture involved in either ethylene synthesis or pectin degradation [110–112]. *PG1* was mapped to chromosome 10 and colocalized with a major hot spot QTL associated to several fruit texture subphenotypes [110]. Sun *et al*. [20] found that mostly *M. domestica* cultivars with crispy fruit texture were heterozygous for this locus whereas many *M. sieversii* accessions were homozygous for the mealy type alleles. Alleles of this major gene associated with measurable differences in fruit texture are highly conserved across the genus [20]. Markers have been developed which can be used for marker assisted selection of *M. sieversii* accessions with a more palatable, less mealy texture [20, 113]. *M. sieversii* and *M. sieversii–M. domestica* hybrids have a large variation in fruit size (Fig. 1) highly similar to domesticated apples. Liao *et al*. [102] found the genes *fs4.1*, *fs15.1*, *fs15.2* all contribute to the size of the fruit in *M. sieversii*. Additionally, Duan *et al*. [19] found that *fw1* and *fw2* [114] are the underlying genes controlling fruit weight conserved across *M. domestica* and *M. sieversii* found on chromosomes 15 and 8, respectively.

## Breeding for resistance to pests and diseases

### Fire blight (*Erwinia amylovora*)

Extensive research into the most destructive apple disease, fire blight caused by *E. amylovora*, has determined *M. sieversii* is an excellent source of resistance [18, 34, 73, 74, 115–118]. The natural incidence of infection was documented on 1151 seedlings planted in 1997 and 1998 at USDA-ARS-PGRR in Geneva, NY [34], as well as on 1410 accessions planted in 1998 in MN [116]. In total, 124 families were evaluated, with 32 families represented at both sites. Only a low incidence of fire blight susceptibility was observed in 12 families, and a total of 535 accessions were classified as highly resistant or resistant [115]. In New Zealand, an evaluation of 936 seedlings from 52 families revealed that only 13% exhibited signs of fire blight infection during natural incidence assessments [119]. In addition, the USDA *Malus* repository in Geneva, NY was evaluated after a large fire blight outbreak in 2020, where 1142 trees of 41 *Malus* species were assessed for average severity of young shoots infected [117]. The majority of the 95 *M. sieversii* and *M. sieversii–M. domestica* hybrid [35] trees evaluated had an average severity score under 10% [117]. Though many of these reports of fire blight resistance come *via* field incidence observations, natural escape, or lack of interaction with the pathogen can commonly misclassify resistant individuals. For example, 286 potentially resistant *M. sieversii* and *M. sieversii–M. domestica* hybrid [35] seedlings out of the 2590 screened by natural incidence 60% displayed consistent severity scores with controlled inoculations in the greenhouse [115].

Controlled phenotypic screenings in multiple environments with clonal replications have been the preferred method to reliably identify fire blight *M. sieversii* accessions [73]. Harshman *et al*. [35] conducted phenotypic evaluations of nearly 200 *M. sieversii* and *M. sieversii–M. domestica* hybrid accessions using controlled field inoculations in Washington and West Virginia, as well as greenhouse trials in New York. These screenings led to the discovery of 12 accessions exhibiting fire blight resistance levels comparable to the highly resistant control, “Robusta 5” [73]. Several of these accessions have been chosen for incorporation into the apple breeding program at Washington State University [73]. Furthermore, in the Swiss breeding program, 12 fire blight resistant *M. sieversii* and *M. domestica–sieversii* hybrid genotypes were identified with controlled field inoculation and selected for further crosses in 2010 [74].

There are currently few known fire blight resistance QTL from *M. sieversii* that have been identified, highlighting the need for more genetic mapping studies. Desnoues *et al*. [120] identified 13 novel strain- and environment-specific minor QTL linked with fire blight resistance from the cross of *M. domestica* cv. “Royal Gala” × *M. sieversii* “KAZ 95 18-07”. Recently, a moderate-effect fire blight resistance QTL (*Msv_FB7*) on linkage group 7 was identified from the paternal parent of the cross “Royal Gala” × *M. sieversii “*KAZ 95 17–14” [118]. *Msv_FB7* explained about 48–53% of the phenotyping variance and molecular markers were developed to utilize this QTL for marker-assisted selection [118]. *Msv_FB7* shows potential to be a useful source of fire blight resistance to develop fire-blight-resistant cultivars with less generation time than other wild species of apple [118].

### Apple scab (*Venturia inaequalis*)

**Figure 3:**
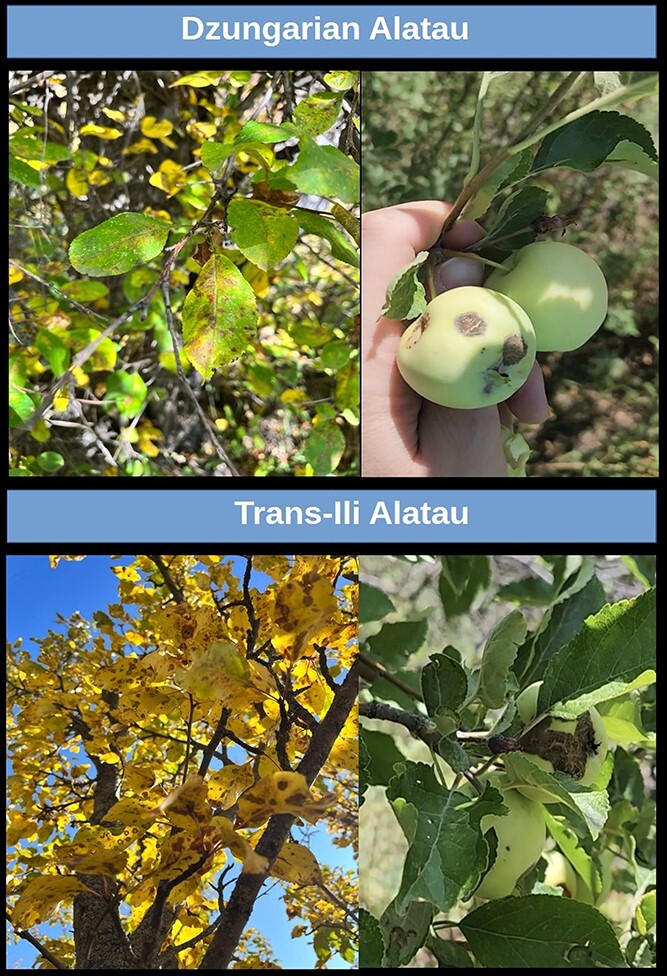
Images of apple scab symptoms on fruit and leaves of wild *Malus sieversii* (Ledeb.) M.Roem trees growing in the Northern Tian Shan mountains ranges Dzungarian Alatau and Trans-Ili Alatau in Kazakhstan.

*M. sieversii* offers an opportunity to become an excellent breeding source for resistance to apple scab, the most economically impactful disease of apples [121, 122]. This apple species is considered the original host of the *Venturia inaequalis* populations currently infecting domesticated apples (Fig. 3) [123, 124]. Populations of *V. inaequalis* found on secluded *M. sieversii* plants in mountains from Kazakhstan represent ancestral relict of the current agricultural and urban Central Asian and European *V. inaequalis* populations [124]. The evolutionary divergence between the ancestral *V. Inaequalis* population and other Central Asian and European populations has occurred during an estimated period ranging from 2000 to 4000 bp [124]. It did not lead to speciation, but the resistance between host populations could nonetheless be affected. This hypothesis is consistent with the high levels of variance in resistance to apple scab observed among *M. sieversii* accessions inoculated with *V. inaequalis* isolates from different geographical areas [125].

Evaluations of various germplasm collections and biparental seedling populations for scab resistance indicated that in selected accessions of *M. sieversii* and *M. sieversii–M. domestica* hybrids [35] and show significant levels of resistance [18, 34, 126–131]. Inoculation with seven *V. inaequalis* strains of 3000 seedlings from 220 wild *M. sieversii* and *M. sieversii–M. domestica* hybrid [35] trees across 10 diverse ecosystems in Kazakstan, Uzbekistan, Kyrgyzstan, and Tajikistan over a 6-year evaluation period indicate the presence of scab resistance in this species [126]. Chlorosis with crinkling, stellate necrosis, or extended necrosis were observed 2–4 weeks after inoculation in more than 20% of the seedlings [126]. The resistance of individual populations ranged from 0 to 75% of seedlings. Similarly, Forsline and Aldwinckle [34] observed scab resistance in 40% and 47% of seedlings among 388 and 204 seedlings at two collection sites in Geneva, NY. Later on, Fazio *et al*. [115] tested 1480 seedlings in Geneva, NY and resistance were recorded in 41% of the seedlings that was confirmed in more than half of the originally resistant grafted plants. This was repeated on grafted plants, and reactions resembling reactions associated with *Rvi8* and reactions similar to the *Rvi15* gene showed 100% agreement with the genotype, and those similar to *Rvi6* agreed in 50% of evaluations. In New Zealand, researchers evaluated over 1400 *M. sieversii* trees belonging to 52 seed lots and observed low heritability (0.13 on a family mean basis) as a result to high levels of scab resistance to natural field infection [18].

Based on these observations, gene-for-gene resistance has been analyzed for various *M. sieversii* accessions [123, 128, 131], indicating four yet uncharacterized scab resistance genes in this species [18]. *Rvi8* on linkage group 2 is the main scab resistance gene that confers resistance to majority of *V. inaequalis* strains was identified in *M. sieversii* open-pollinated population GMAL 3631-W193B from the Tarbagatai mountain range in Kazakhstan [128]. Upon inoculation with *V. inaequalis* race (8) isolates NZ188B.2 [128] and 1639 [127], stellate necrotic reaction has been associated with the GfG relationship. *Rvi8* co-locates with *Rvi2* from Russian apple R12740-7A on linkage group 2 of apple and may be the same gene, although the former gene seems to be compatible with *V. inaequalis* race 8 isolate, whereas *Rvi2* confers resistance to that race [123]. Furthermore, scab-resistant *M. sieversii–M. domestica* hybrid accession PI 613988 was crossed to “Royal Gala” to create GMAL 4595 population [131]. Inoculation of 188 seedlings from this population with *V. inaequalis* races (1) and (2) and association analysis using 287 simple sequence repeats (SSR) markers resulted in discovery of four scab-resistance loci on the distal end of linkage group 2, i.e., *SNR1, SNR2, ChlR1, ChlR2*. Similarly, to *Rvi8*, the former two loci confer stellate necrosis, while the latter two are associated with chlorotic lesions. It is yet to be demonstrated if *SNR1* and −2 are identical-by-state (IBS) to *Rvi8*, which will require additional inoculation tests using *V. inaequalis* race (8) and functional characterization. Altogether, *M. sieversii* as a breeding parent could alleviate the breakdown of formerly scab-resistant *Rvi6*-harboring cultivars by *V. inaequalis* race 6 [132].

### Blue mold (*Penicillium expansum*)

Quantitative resistance to postharvest diseases was observed in *M. sieversii* and *M. sieversii–M. domestica* hybrids [35] in assessments against blue mold (*Penicillium expansum*) and bitter rot (*Colletotrichum acutatum*) [133, 134]. These diseases cause significant economic damage to apple sector and no major resistance has been discovered so far. Blue mold infection was evaluated among elite accessions from Kazakhstan germplasm collection in Geneva, NY. It resulted in the identification of six resistant accessions, i.e., GMAL 3610.i, 3682.c, 3684.c, 3689.e, 4286.g, and 3614.c [134]. In a multiyear trial [133], six and four consistently resistant GMAL accessions, respectively, 3635.i, 3689.i, 3689.p, 3688.h, 3625.a, and 3547.n, showed blue mold resistance over three years of evaluation, and PI 369855, GMAL 3689.h, 3709.c, and 3690.l were resistant against bitter rot in 2009, and many more showed only moderate resistance to both diseases.

Defense response mechanisms and QTLs conferring resistance to postharvest decay were identified in *M. sieversii* plants. Within the first 4 days upon wounding of a fruit, more resistant plants respond more rapidly to wounding within the first 24 hours and hence prevented the fungus from successfully infecting the tissue [135]. Reactive oxygen species (ROS) were associated with the rapid response, whereas callose and lignin/suberin appear to play a less prominent role [135]. Furthermore, resistant accessions contain higher concentrations of specific phenolic compound groups, including procyanidins, dihydrochalocone, flavonols, and hydroxycinnamic acids [136]. Transcriptomic comparison of blue-mold-resistant to moderately resistant accession *M. sieversii* PI 613981 [133, 134] and susceptible “Royal Gala” confirmed the more rapid response in the resistant genotype to wounding and inoculation with *P. expansum* within the first 48 hours [137]. Ethylene pathway, jasmonic acid pathway, and *MYB* domain transcription factor family genes are differentially expressed in the resistant genotype compared to susceptible one. Accession PI 613981 (GMAL 4593) was used in a cross with “Royal Gala” to identify QTLs associated with blue mold resistance [109]. In 2 years of analysis on 98 individuals, a QTL for blue mold resistance was identified on LG 4 between 30 and 35 cM. Two additional QTLs were identified in the population of 169 individuals from the same family [138]. The QTLs *qM-Pe3.1* and *qM-Pe10.1* mapped between 67.3 and 74 cM on linkage group 3 and 73.6 to 81.8 cM on linkage group 10 accounted for 27.5% and 14% of the experimental variability, respectively. Diagnostic markers for the latter two QTLs are available [103, 138]. Rapid cycle breeding based on the use of markers in combination with crosses of T1190 fast-flowering transgenic line enabled introgression of the *qM-Pe3.1* resistance allele into breeding germplasm [103]. *M. sieversii* germplasm is yet to be evaluated for other postharvest diseases such as gray mold caused by *Botrytis cinerea* or anthracnose caused by *Neofabraea* species.

### Resistance to additional diseases and pests

Accessions of *M. sieversii* and *M. sieversii–M. domestica* hybrids showed enhanced resistance levels to several other diseases and pests, including powdery mildew, cedar apple rust, replant disease, canker, woolly apple aphid, and apple maggot [18, 139–141]. However, compared to scab, fire blight, and blue mold, research on resistance to other diseases of *M. sieversii* is substantially more limited. Firstly, the majority of young *M. sieversii* seedling plants in Germany and New Zealand show susceptibility to powdery mildew (*Podosphaera leucotricha* (Ell. And Ev.) E.S. Salmon), and progressively develop ontogenic resistance with maturation [18]. Only 70% and 45% of the seedlings showed mildew 2 and 3 years after planting, respectively, compared to the young plants. Secondly, cedar apple rust (*Gymnosporangium juniperi–virginianae* Schwein.) resistance was observed in 55% of 1480 seedlings in New York [115], whereas ~30% of the 3000 seedlings in New York and New Jersey were resistant [18]. Thirdly, some *M. sieversii* accessions show promising resistance to apple replant disease as well. *M. sieversii var. sieversii f. niedzwetzkyana* “MAL0970” [140] and *M. sieversii* accessions PI 600427 and PI 600563 [139] showed substantial tolerance to apple replant disease. Their growth in soil contaminated with replant pathogens, such as *Pythium, Cylindrocarpon, Fusarium, Rhizoctonia*, and *Phytophthora*, was comparable to the growth observed for plants grown in irradiated soil [140]. Finally, apple canker disease can severely affect tree growth and root development [142]. Field inoculations of branches on 28 different *M. sieversii* accessions with five pathogenic isolates of *Botryosphaeria dothidea* at Quzhou Experimental Station of China Agricultural University demonstrated that a wide range of canker resistance exist in this species [141]. Defense response to Valsa canker infection is based on early infection jasmonate (JA) pathway activation during the first 3 hours upon inoculation [143]. At later infection stages, jasmonate activation is attenuated followed by activation of salycilic acid pathway, from 3 to 6 hours upon inoculation. This response is largely based on differential expression of genes encoding transcription factors (e.g., *WRKY*), plant–pathogen interaction proteins, plant hormone signal transduction proteins, flavonoid biosynthesis proteins, and phenylpropanoid pathway proteins [143]. Furthermore, differentially expressed gene families in *M. sieversii*, including *MYB* transcription factors gene family, Basic/helix–loop–helix (*bHLH*) family, and chitinases were functionally validated [144–146]. *MsMYBs*, *MsMYB14* and *MsMYB78* [145], *MsChi35*, a class IV chitinase [144], *MsbHLH155.1* [146] genes all can reduce susceptibility when transiently overexpressed in *M. sieversii*.

**Figure 4:**
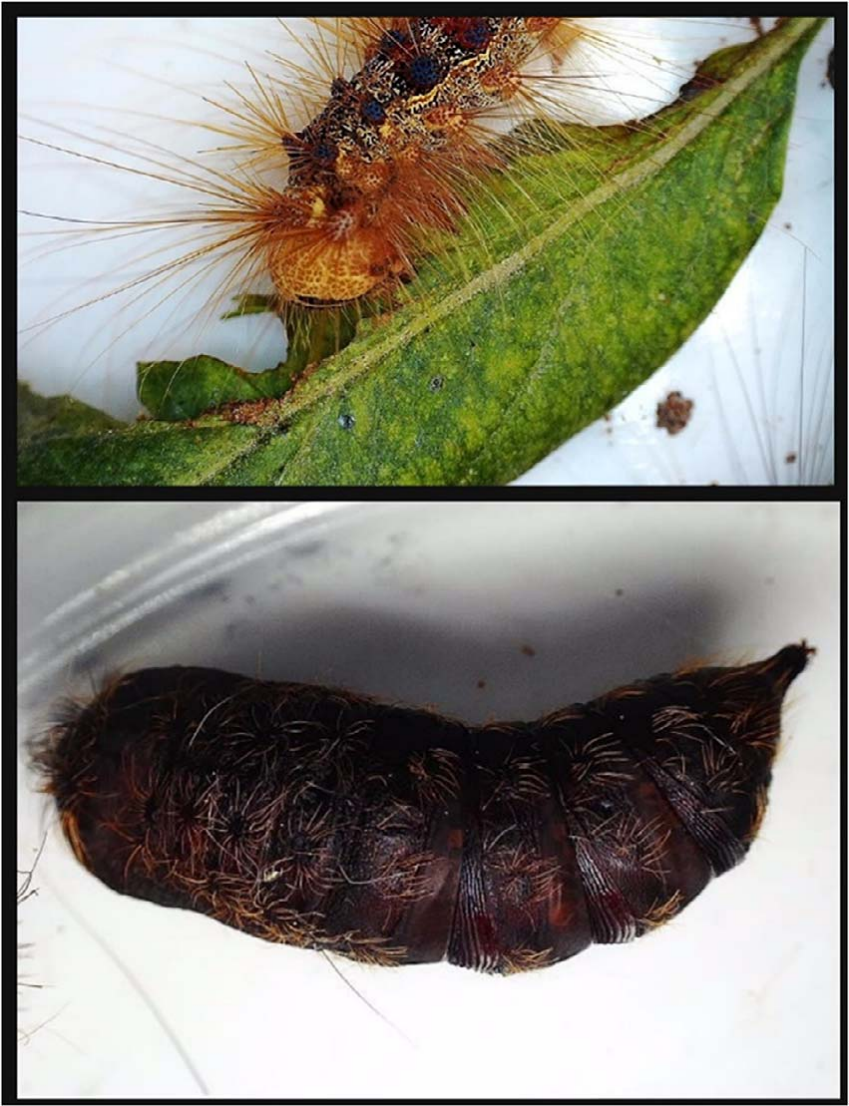
Spongy moth collected in wild *Malus sieversii* (Ledeb.) M.Roem populations of Trans-Ili Alatau in 2023. The top panel represents the caterpillar of *Lymantria dispar*, while the pupa is depicted in the bottom panel.

Virus-free plant germplasms are critical for successful growth, distribution, and breeding of *Malus* genotypes [147]. Different viral species are present among diverse range of *M. sieversii* host accessions, including apple chlorotic leaf spot virus (ACLSV) and apple stem grooving virus (ASGV), and apple stem pitting virus (ASPV) [147–149]. In Kazakhstan, for instance, ACLSV affected 53.8%, ASPV 30.8%, and ASGV 5.1% of *M. sieversii* and *M. domestica* accessions. Cryotherapy enabled generating virus-free shoots in over 60% of plants [147].

Populations of *M. sieversii* are declining partially due to damaging insect pests [150, 151]. In Kazakhstan, arachnids and insects, such as codling moth (*Cydia pomonella*), the rose tortrix (*Archips rosana*), apple leaf skeletonizer (*Choreutis pariana*), European red mite (*Panonychus ulmi*), gypsy moths formally known as spongy moths (*Lymantria dispar*), and apple buprestid (*Agrilus mali*), are considered the most notorious threat to *M. sieversii* preservation [150, 152, 153]. Notably, the spread of the spongy moths among populations of wild apples in Kazakhstan has increased over the past 3 years (Unpublished data, Fig. 4). Identification of pest-resistant and -susceptible hosts can aid identification of genes and defense mechanisms involved in pest resistance of *M. sieversii* and enable breeding of resistant cultivars [154]. So far, *A. mali* resistance identification and characterization was conducted by studying infestation defense response in resistant and susceptible *M. sieversii* accessions in Xinjiang Uyghur Autonomous Region, China. This pest has currently damaged 95% of wild *M. sieversii* forests in area [153]. Resistant trees contain increased phenolic and tannin contents and are low in soluble sugars compared to susceptible plants [154]. Transcriptomic and metabolomic analysis indicated that resistant plants contain enhanced signal transduction pathway of plant hormones and synthesis of compounds such as terpenes, quinones, flavonoids, and jasmonic acid, resulting in higher contents of trans-cinnamic acid, caffeine, and ferulic acid after infestation [154]. In New York, apple maggot (*Rhagoletis pomonella* Walsh) survival was analyzed in *M. sieversii* seedlings and “McIntosh” fruits [18]. Although all fruits showed infestation, fruits of *M. sieversii* seedlings were 3–94% less infested than the “McIntosh” fruit, as has been reflected in apple maggot larvae survival rate. Resistance to spider mites, red mites, brown apple moth, woolly, rosy, and green aphids was suggested to show genetic variation in *M. sieversii* and require further dedicated studies [18].

## Breeding for abiotic stress tolerance

Long periods of extreme environmental disturbance of tree growth result in degeneration of wild fruit forests [155]. Abiotic stress, including drought, osmotic, and frost stress all have adverse effects on plant growth and populations of *M. sieversii* [155–159]. A comprehensive study on drought tolerance of various *M. sieversii* accessions is lacking, but rootstocks of *M. sieversii* are considered to have beneficial drought- and osmotic stress-tolerant characteristics [18]. Identification of specific genotypes tolerant to abiotic stressors and genes and molecular mechanisms that play a role herein is vital to efficiently utilize diverse *M. sieversii* germplasm in breeding for abiotic stress tolerance. Leaf characteristics, such as area, length, width, and stomatal density vary largely in *M. sieversii* populations and were suggested to affect overall drought tolerance of the trees [156, 158]. Similar to leaves, roots may play a vital role in drought tolerance as well. Rootstocks of *M. sieversii* compared to the R3 rootstock performed better in drought [157]. Enhanced transcription regulation, hormone biosynthesis, peroxidase activity, superoxide dismutase activity, and abscisic acid (ABA) content was found in *M. sieversii* under drought stress, while contents of auxins were lower compared to R3 [157]. Also, microRNA 156ab [160], gene *MsUspA* [161], dehydration-responsive element-binding factor 2 (*DREB2*) [162], Nuclear factor Y (*NF-Y*) [143], and protein kinases [163] are upregulated in *M. sieversii* in response to drought stress, affecting various molecular mechanisms including increased antioxidant enzyme activities, proline accumulation, and ABA accumulation. Furthermore, inhibition of *miR164g* in *M. sieversii* and enhanced expression of *MsNAC022* alleviates drought stress and lays a foundation for breeding drought-tolerant plants [164].

Contrasting osmotic stress and drought/heat tolerance, cold hardiness and tolerance to frost damage is highly desired in apple growing in temperate climate regions at high latitudes [18]. Different wild populations of *M. sieversii* in China show different levels of cold hardiness, whereas the difference is smaller within populations [165]. Cold hardiness determined by means of the electrical impedance spectroscopy (EIS) method is higher in populations Gongliu compared to Xinyuan populations. During cold acclimation of leaves from *M. sieversii* seedlings, jasmonate, indole-3-acetic acid, and abscisic acid their contents increased, and they decreased during freezing stress [159]. Mechanisms involving plant hormone signal transduction, starch, and sucrose metabolism, peroxisomal activity and photosynthesis were enriched and transcription factors *DREB1/CBF, MYC2, WRKY70, WRKY71, MYB4,* and *MYB88* were strongly induced during stress period and might play a role response of *M. sieversii* to freezing stress. Conversely, 12 genes encoding heat shock proteins linked with heat tolerance have been identified from *M. sieversii* [166]. One gene *MsHsp16.9* on chromosome 7 was confirmed to underpinned tolerance to heat stress through functionally validated with an *Arabidopsis* homolog [166].

## *In situ* and *ex situ* conservation of *M. sieversii* in a changing climate

Wild standings of *M. sieversii* across Central Asia are important genetic reserves that also provide many ecosystem services in that region [40]. These wild apple forests have been in steady decline, especially in their natural habitat near Almaty, Kazakhstan [12]. In total, 29 *Malus* species including *M. sieversii* are listed as vulnerable in the threatened species red list, according to the International Union for Conservation of Nature (IUCN) [11, 167]. Most of these species are classified as data-deficient because there is too little information for the accurate assessment of the conservation status. In 1981, *M. sieversii* and *M. niedzwetzkyana* were added to the “Red Book of Kazakh SSR”, a comprehensive guide to the threatened species of Kazakhstan [168, 169]. More updated guides outlining the vulnerable state of *M. sieversii* have since been released from the IUCN [11, 167, 168]. Although there are areas that *M. sieversii* populations are not threatened such as Kyrgyzstan [170], this species is considered in a vulnerable state given several natural and anthropogenic factors. Some the major factors affecting these wild standings are (1) pests and diseases (2) crop-to-wild gene flow from locally planted *M. domestica* cultivars (3) human-driven habitat fragmentation, degradation, and urbanization and (4) climatic shifts in the optimal apple growing conditions.

### Pests and diseases

Among the greatest factors contributing to the decline of wild apple trees in Central Asia is the increasing pressure of pests and disease [171, 172]. The most devastating disease of apples, fire blight, is not native to the region and was first registered in 2008 [171]. The arrival of this pathogen was likely attributed to large imports of plant material from Europe [173]. From 2008 to 2015, a large import of seedlings and stock of apples, pear, and quince was brought in by the government to bolster fruit production [173]. These are all rosaceous fruit crops that can contract and harbor fire blight, thus introducing fire blight to these regions [173]. This led to the spread of fire blight across the Amaty fruit zone in only several years [174–176]. One danger of a newly emergent plant pathogen is the unknown level of genetic resistance among the wild standings given *M. sieversii* did not coevolve with *E. amylovora* [17, 177]. From these damaging outbreaks emerged fire blight monitoring and prevention program led by Kazakhstan [171]. These efforts include enforcing laws for the import of clean apple stock, government funded management practices, and programs to pay growers for removing infected trees [171].

In Kazakhstan, arachnids and insects, such as codling moth (*Cydia pomonella*), the rose tortrix (*A. rosana*), apple leaf skeletonizer (*Choreutis pariana*), European red mite (*Panonychus ulmi*), spongy moth (*L. dispar*), and apple buprestid (*Agrilus mali*), are considered the most notorious threat to *M. sieversii* preservation [150, 152, 153]. Jashenko *et al*. [152] found the three most prevalent defoliating insects affecting *M. sieversii* in the Trans-Ili Alatau mountain range are the apple ermine moth (*Y. Malinellus Zell.*), the rosebush leaf roller (*A. rosana L.*), and the hawthorn leaf roller (*C. crataegana Hb.*). Among the largest threats is the wood-boring beetle, *Agrilus Mali*. In the past several decades, *A. mali* has damaged an estimated 40% (3866.67 hm [2]) of the area of wild apple forest in Tianshan and killed 666.67 hm [2] since the first detection in 1993 [178, 179]. This pest is currently damaging to 95% of wild *M. sieversii* forests in the Xinjiang Uyghur Autonomous Region of China [153]. Moreover, the vulnerability of these damaged wild apple trees increases secondary infection of the fungal pathogen, Valsa canker, *Valsa mali* var. *mali*, which can accelerate tree mortality [155, 180].

### Reciprocal gene flow

The genetic erosion of *M. sieversii* is one of the greatest factors undermining conservation efforts of the vulnerable wild standings in Central Asia [12]. Since the advent of more accessible DNA sequencing and marker technology, crop-to-wild introgression and admixture can be more accurately estimated [35, 63]. Several studies have found increasing threats to the genetic integrity of *M. sieversii* in protected forests due to geneflow with *M. domestica* grown in adjacent areas [9, 35, 50, 169]. Kazakhstan’s Zoning laws in the 1960’s expanded the use of private land surrounding the wild apple standings [169]. More cultivated apples were planted in these private gardens which would then hybridize with local wild trees [169]. Between 1932 and 1967, wild *M. sieversii* apple trees were often used as rootstocks for cultivated apples increasing the chances of hybridization [169]. Private gardens adjacent to the wild forests, along with cultivated apples planted within them, create large reservoirs of pollen near the natural stands of *M. sieversii* [169]. This opened avenues for reciprocal gene flow between *M. sieversii* and *M. domestica* as pollen from these trees can be carried by pollinators up to 10.7 km [181]. Consequently, the natural systems of wild apple forests in Kazakhstan lost species-specific dynamic features and genetic integrity [169]. As each generation is increasingly admixed with these cultivars these become less adapted to the local environment and require more resources to manage *in situ* [12]. Additionally, the increased fruit size of admixed hybrids reduces the means of dispersion making *M. sieversii* wild standings often grown in tight clusters [32]. Large and carefully selected *in situ* reserves could conserve the genetic diversity in wild populations of *Malus* species, if effectively managed [12]. Omasheva *et al.* [169] recommended the high elevation sites at Krutoe truct and Tauturgen in Kazakhstan as nearly no admixture or reciprocal gene flow with *M. domestica* has been observed. Volk *et al*. [35] found that the 12 accessions derived from Kyrgyzstan they studied were all pure *M. sieversii* and accessions from the Karatau region in Kazakshtan showed very low rates of admixture with *M. domestica*. Local governments could this knowledge to enact exclusion zones and prevent further cross contamination and hybridization with *M. domestica* within a certain range [12].

### Habitat degradation

The habitat of *M. sieversii* is at risk of loss due to the encroachment of agricultural land, livestock overgrazing, urban development, and firewood harvesting [34, 168]. Hokanson *et al*. [17] estimated that the apple forests in Kazakhstan suffering from human encroachment lost about 90% of the wild apples that existed near Almaty in 1935. Eastwood *et al*. [168] concluded the damage to these forests has resulted in the reduction of total area to 7% of the area recorded in 1930 and 70% of that decline happened during the last 30 years. In the late nineteenth century, pressure began on the wild apple forests of Zailiysky Alatau locals were clear cutting forests in the mountain foothills to plant agricultural crops [182]. However, in many forest tracts, these management and protection rules are not adhered to [170], leading to extensive environmental damage [183]. The ability for natural renewal of remaining wild stands of apple were weakened as the number of young saplings was greatly reduced compared to mid- to older age trees [184]. Changes in land zoning laws in the 1960s and 1970s and the new Land Code in 2003 approved privatization of this land [169]. This contributed to a loss of biodiversity in these regions due to the use of plots for private gardens in the mountainous areas being permitted [169]. Additionally, Zhang *et al*. [153] described how aridification across Central Asia further contributes to genetic isolation of *M. sieversii* populations. These natural barriers prevented admixture of populations from the Chinese western Junggar Mountains, Dzungarian Alatau in Kazakhstan and Tajikistan, Talas Alatau from Kazakhstan to Kyrgyzstan, and Ili Valley in China and Kazakhstan [185]. Similarly, the large reduction of wild apple forests in Kyrgyzstan is due to unsustainable firewood harvesting and unrestricted livestock grazing driven by the most recent economic recession [186]. Management practices have been proposed to help alleviate the effects of this land fragmentation [187]. Instead of cutting down forest for grazing, Xu *et al*. (2022) recommended that cattle grazing be allowed among wild *M. sieversii* forests as it both reduces weed competition and effectively contributes to apple seed dispersal. However, though older apple trees benefit, Jia *et al.* [146] showed with remote sensing data that grazing negatively affects *M. sieversii* trees less than 4 years olds and is causing great harm. The most important sites of these wild forests in Kazakhstan and Kyrgyzstan have had management strategies implemented to achieve sustainable use of forest resources [188, 189].

### Climate change

The intensification of climate change in the center of origin of the domesticated apple has made conservation efforts exceptionally difficult [190]. A 70-year climate change analysis in Kazakhstan revealed that the average annual temperature increased by 0.28°C year^−1^ [191]. Moreover, the maximum recorded warming occurred in winter, annual precipitation showed a weak downward trend, and variability in temperature extremes greatly increased between 2000 and 2011 [191]. These striking changes have made it more difficult to grow even resilient staple crops across Kazakhstan including potatoes, wheat, and barley [192, 193]. Panyushkina *et al*. [40] found negative growth patterns of wild apples in the Lake Balkhash Basin of Kazakhstan are driven by unprecedented and intensified Arctic Oscillation in winter–spring time after the late 1970s. Current climate models estimate by 2050 the center of wild apple distributions will need to move ~200 m higher in elevation and ~ 160 km northward and to keep pace with the rate of climate change in the region [194]. This loss of viable *in situ* preserve sites may involve implementing more drastic measures, such as the assisted migration of native apple populations [169, 194]. Additionally, environmental shifts due to climate change can also accelerate the emergence of new strains of existing pathogens that threaten apples [66]. Efforts to protect *M. sieversii* habitats are important safeguard biodiversity against direct human impacts [194].

### *Ex situ* preservation

Genebanks play a crucial role in the long-term preservation of genetic material essential for human agriculture [12, 17]. To maintain apple diversity, 35 genebanks currently hold a total of 33 588 *Malus* accessions, which include seeds from local and international cultivars as well as wild *Malus* species such as *M. sieversii* [190]. Nearly all these accessions are cultivated *ex situ* as grafted trees in the field [190]. The oldest and most centralized *ex situ* collection of *M. sieversii* was established by Aimak Dzangaliev in Kazakhstan [195]. Dzangaliev, a former student of Vavilov, spent many years selecting valuable genotypes of *M. sieversii* in Tian Shan before any USDA expeditions [195]. Over 20 genotypes of genetically pure wild *M. sieversii* selected by Dzangaliev are planted in the National Botanical Garden [41, 196]. Several Kazakhstani collections of *M. sieversii* are established *in situ*. Additionally, several *in situ* collections of *M. sieversii* have been established in Kazakhstan, with the most significant being the genetic reserves in Dzungarian and Trans-Ili Alatau, which have an approximate average age of 30 years [17].

Many genebanks worldwide compile and retain detailed information about their collections of wild apples from Central Asia [190]. Currently, the USDA Agricultural Research Service’s (ARS) National Plant Germplasm System (NPGS) maintains a collection of over 363 accessions of *M. sieversii* and *M. sieversii–M. domestica* hybrids [36]*.* The four expeditions to collect this material were conducted from 1989 to 1996, targeting 12 sites across Kazakhstan, Kyrgyzstan, Tajikistan, and Uzbekistan with unique climatic conditions [34]. Taxonomic, phenotypic, and passport data of these accessions are maintained on databases such as the USDA Germplasm Resource Information Network (GRIN)-Global database, European Search Catalogue for Plant Genetic Resources (EURISCO), and Genesys [36, 197, 198]. The Genome Database for Rosaceae (GDR) is a comprehensive collection of genomic resources, molecular marker data, and analytical tools to empower breeding and genetics research [199, 200]. Several other organizations collect and disseminate knowledge of diverse apples, including the ECPGR Malus/Pyrus Working Group, the People’s Trust for Endangered Species (PTES), and Orange Pippin. The conservation, characterization, evaluation, and distribution of apple genetic resources relies on these groups to support breeding programs and public use [197, 201–203].

**Figure 5:**
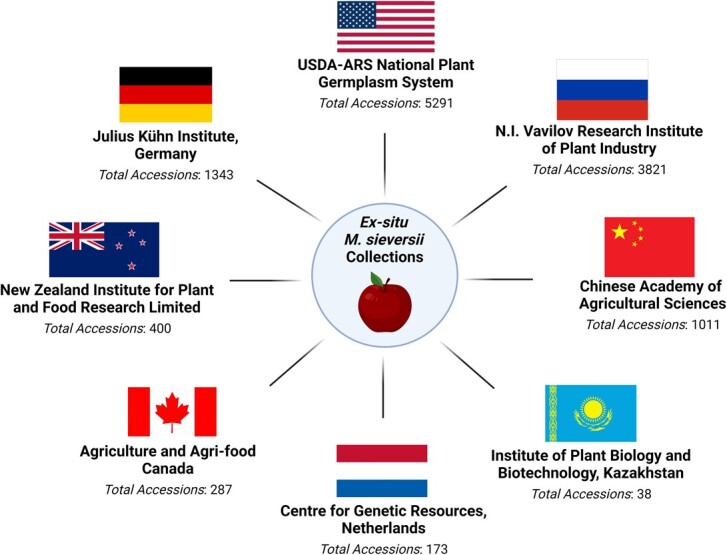
A graphical representation of the major national programs that participate and contribute to the *ex situ* conservation and research of *Malus sieversii* (Ledeb.) M.Roem. Below each program name is the number of total *Malus* accessions maintained in each program sourced from Bramel and Volk [190].

The distribution of vital germplasm material by genebanks enhances the breeding and conservation of apples [12]. Apple germplasm material can be distributed as budwood, leaves, pollen, fruit, seeds, and sometimes grafted trees [12, 190]. The exchange of apple germplasm material internationally can be challenging due to unique phytosanitary regulations across different countries [190]. International shipping of plant material can require phytosanitary certificates and several inspections before approval to enter a country [190]. These strict rules aim to disrupt the transport of material suspected of harboring quarantined pests and pathogens that can spread globally [190]. Meeting these standards can mean several years of cleaning and testing plant material before approval [190]. These efforts aim to protect *Malus* diversity and are coordinated across many national agricultural programs, including those in the United States, Russia, China, and many more [190] (Fig. 5).

Gene banks use several primary methods for preserving *M. sieversii*, including *ex situ* plantings, *in situ* preserves, cryopreservation, and tissue culture [12]. *E -situ* collections of *M. sieversii* are among the best methods for preserving diverse genetic material [8]. These collections allow material for grafting and distribution to be maintained and made available for research and breeding efforts simultaneously [4, 8]. Though having a national genebank improves the standardization of accession naming and phenotyping protocols, there is a need to better standardized protocols across international genebank programs [12]. Compared to *in situ* preserves, controlled field plantings enable more efficient maintenance and better standardization of genotype information [4, 8]. However, vegetatively propagated collections are the most expensive to maintain among the various preservation methods [12]. These collections require year-round maintenance, including pruning, replacing trees, and regular, costly applications of pesticides and herbicides [12]. Since these trees are planted outside their native habitats, there is a risk of losing important genotypes to various biotic and abiotic stressors [117]. Curators of *ex situ* collections must also consider the ethical implications of using germplasm material, particularly how it affects the communities of origin [204]. To ensure the equitable use of germplasm, it is crucial to foster collaborations with the communities and institutions associated with these collection sites [12, 204]. This approach ensures that the benefits are distributed equitably, providing advantages to all stakeholders instead of favoring a particular group [204]. These collections are intended to serve public interests and are often created for various purposes, including use in breeding programs, long-term conservation, and the distribution of budwood or grafted trees [7, 12, 44, 45, 101].

### *In situ* preservation

There are many ongoing efforts to protect wild *M. sieversii* apple species, *in situ*, in their native habitats [169, 190, 194]. *In situ* conservation projects to protect ancient apple forests have been identified in Azerbaijan, China, Kyrgyzstan, and Kazakhstan *via* designated nature preserves [41, 190]. Though *M. sieversii* forests were common across Central Asia, the remnants of these forests now exist primarily in Kyrgyzstan, Xinjiang region of China, and the Trans-Ili and Dzungarian (previously named Zailiiski and Djungarskii) mountains of eastern Kazakhstan [12, 41]. Efforts of these countries to protect these wild fruit forests include legally protected forest areas, participatory (collaborative) forest management, and programs to monitor and manage pests and diseases [170–172, 189, 194]. However, it is challenging for these countries to maintain the wild apple populations given the mountainous terrain, fragmented distribution of the forests, and management restrictions of specially protected natural territories [171, 172]. Moreover, these protections put in place are not often adhered to which can be tied to economic conditions of locals [189]. This is prevalent with the apple forests in Kyrgyzstan where apple trees are still foraged for fire wood and the land is used for livestock grazing [189, 205]. Enforcement of existing conservation efforts in wild apple forests of Kyrgyzstan and Kazakhstan are important particularly for the regions protected for admixture with *M. domestica* [35]*.* International collaboration on conservation, conservation research, documentation, or phenotyping is also currently limited [190]. Further bolstering this international collaboration could accelerate the transfer of knowledge and resources to protect these genetic reservoirs more effectively [190].

### Cryopreservation

The most common method for long-term preservation of apple tissue is the use of liquid nitrogen or liquid nitrogen vapor to halt the metabolism and division of the cells in the tissue [206]. Cryopreservation is the method of using extreme cold (−165°C to – 196°C) to maintain living apple tissue in long-term storage to be later regenerated [207]. There are simple and efficient protocols available for the cryopreservation of pollen, seeds, *in vivo* dormant buds and in vitro shoot tips [208–212]. *In vitro* shoot tip cryopreservation is particularly useful given it can produce virus free tissue for clean propagation [207]. Maintaining genetic material this way is still a challenging task as cryopreservation programs require specialized equipment and a costly supply of liquid nitrogen [213]. There have been many efforts to utilize this method to cryopreserve *M. sieversii* accessions for either grafting or tissue culture. Kushnarenko *et al*. [214] characterized the effects of cryopreservation in over 4400 seeds from 34 *M. sieversii* accessions with a 72–90% germination rate. Towill and Bonnart [215] found among 30 *Malus* species tested, *M. sieversii* was among the most tolerant to bud cryopreservation with an 86% survival rate across 12 accessions. Volk *et al*. [216] showed that among the 99 *M. sieversii* accessions flagged to have buds cryopreserved by the USDA-ARS National Center for Genetic Resources Preservation the survival rate ranged from 58 to 77%. These efforts have already proven to be useful backups as devastating diseases such as fire blight continue to threaten *ex situ* germplasm collections [117]. This method provides a very valuable opportunity for apple breeding and conservation programs to maintain important genetics critical to the preservation of the species.

### Tissue culture

Preservation and cloning of *M. sieversii* accessions *via* tissue culture is a method of sterile proliferation and regeneration of plant material in a nutrient dense gel medium. Apple tissue can proliferate via shoot or callus culture. This method is important for generating disease free replications of a certain accession in large quantities. Additionally, meristematic tissue from apple in vitro shoot tips can be replicated faster and at higher quantity compared to other methods. Tissue culture media recipes are often genotype specific, though there are protocols available for several *M. sieversii* genotypes [217, 218]. However, there are several drawbacks to using this method to preserve important genetic material. Firstly, tissue culture incurs the risk of accumulating mutations, structural variations among other somaclonal variations [219]. Next, the genotype specificity makes it challenging to preserve a large set of accessions. Also, specialized facilities, equipment and reagents are required that are expensive to purchase and maintain [219]. Tissue culture is more commonly used for transgenic and genome editing experiments to better understand molecular mechanism rather than a method to preserve genetics. However, it is useful to quickly replicate a small set of important genotypes and preserve meristems through either cryopreservation or regenerated and rooted for growth in the field [207].

## Conclusions and future prospects

*M. sieversii* is the most important progenitor species to the development of the domesticated apple and an invaluable source of beneficial alleles for future breeding efforts. Large-scale cultivation of a narrow set of apples leaves the industry vulnerable to disruption while facing climate change and rapidly evolving pests and diseases. The plethora of known alleles linked to resistance to abiotic and biotic stressors will enable the breeding of cultivars that can reduce the risk of disruption to commercial production. More research is needed to understand the genetic underpinnings of beneficial traits found across available *M. sieversii* germplasm. Such studies will enable more genetic mapping, marker development, and functional gene validation to accelerate breeding for biotic and abiotic stressors.

The declining status of wild *M. sieversii* standings in Central Asia and Northwest China is a threat to the long-term preservation of this genetic resource and the ecology of the region. The current landscape of habitat degradation, climate change, and disconnect between international gene banks leaves *M. sieversii* in a precarious state. Conservation *M. sieversii* is an intricate multifactorial issue that will require much cross-institutional collaboration. An increase in international-level collaboration to exchange expertise, resources, and germplasm is needed to appropriately address these major issues. Particularly with the institutions and research groups in the native regions with the most connection to the *in situ* preservations of *M. sieversii*. The sustainable preservation of apples as a high value globally consumed fruit hinges on the renewed focus of breeding and conservation of *M. sieversii*.

## Declarations

Not applicable.

## Supplementary Material

Web_Material_uhae244

## Data Availability

Not applicable. This is a review article and there is no additional data. The data that support the conclusions and statements are included in the article.
