# Supplementary material for: Malus sieversii: a historical, genetic, and conservational perspective of the primary progenitor species of domesticated apples
Source: Hortic Res. 2024 Aug 30;12(1):uhae244. doi: 10.1093/hr/uhae244 (PMC11718403; doi:10.1093/hr/uhae244)
Supplement: Web_Material_uhae244 [file web_material_uhae244.zip › Supplementary Table 1.docx]

| **USDA PI** | **USDA GMAL** | **Accession ID** | **Species** | **Country** | **Year** |
| --- | --- | --- | --- | --- | --- |
| PI 650994 | GMAL 4781 | MN 80-15-11 | Hybrid | Kazakhstan | 2007 |
| PI 650964 | GMAL 4751 | GMAL 3632 MN 85‐26‐18 | Hybrid | Kazakhstan | 2007 |
| PI 650951 | GMAL 4738 | GMAL 3797 MN 85‐22‐81 | Hybrid | Kazakhstan | 2007 |
| PI 629318 | GMAL 3360.f | USSR-89-35-01 | Hybrid | Kazakhstan | 1989 |
| PI 651006 | GMAL 4793 | GMAL 4039 MN 80‐15‐30 | Hybrid | Kazakhstan | 2007 |
| PI 650983 | GMAL 4770 | GMAL 3689 MN 88‐30‐42 | *M. sieversii* | Kazakhstan | 2007 |
| PI 650981 | GMAL 4768 | GMAL 3683 MN 88‐29‐107 | *M. sieversii* | Kazakhstan | 2007 |
| PI 656998 | GMAL 3541.l | KAZ 93‐12‐02 | *M. sieversii* | Kazakhstan | 1993 |
| PI 657082 | GMAL 4002.g | KAZ 95 18‐02P‐33 | *M. sieversii* | Kazakhstan | 1995 |
| PI 629299 | GMAL 3338.c | USSR-89-32-23 | *M. sieversii* | Kazakhstan | 1989 |
| PI 657753 | GMAL 4818 | DM 9 | *M. sieversii* | Kyrgyzstan | 2009 |
| PI 657756 | GMAL 4821 | DM 29 | *M. sieversii* | Kyrgyzstan | 2009 |
| PI 657758 | GMAL 4823 | DM 32 | *M. sieversii* | Kyrgyzstan | 2009 |
| PI 657762 | GMAL 4827 | DM 46 | *M. sieversii* | Kyrgyzstan | 2009 |
| PI 657759 | GMAL 4824 | DM 33 | *M. sieversii* | Kyrgyzstan | 2009 |
| PI 657754 | GMAL 4819 | DM 10 | *M. sieversii* | Kyrgyzstan | 2009 |
| PI 657763 | GMAL 4828 | DM 49 | *M. sieversii* | Kyrgyzstan | 2009 |
| PI 629314 | GMAL 3256.c | USSR-89-08-03 | *M. sieversii* | Tajikistan | 1989 |
| PI 596282 | GMAL 3244.a3 | USSR-89-06-03 | *M. sieversii* | Tajikistan | 1989 |
| PI 629311 | GMAL 3280.h | USSR-89-25-02 | Hybrid | Uzbekistan | 1989 |
| PI 629310 | GMAL 3280.g | USSR-89-25-02 | Hybrid | Uzbekistan | 1989 |
| PI 629297 | GMAL 3275.d | USSR-89-24-09 | *M. sieversii* | Uzbekistan | 1989 |
